# Supplementary material for: From sequence to enzyme mechanism using multi-label machine learning
Source: BMC Bioinformatics. 2014 May 19;15:150. doi: 10.1186/1471-2105-15-150 (PMC4229970; doi:10.1186/1471-2105-15-150)
Supplement: Additional file 2 — Java code of ml2db. Additional file ml2db_code.tar.gz contains the Java source code to run the multi-label machine learning experiments and save the results to database. The code’s Javadoc is included. [file 1471-2105-15-150-S2.zip › additional file 2/ml2db/utils/doc/overview-frame.html]

Overview List


All Classes

## Packages

- cern.colt
- cern.colt.function
- cern.jet.random
- cern.jet.random.engine
- edu.cornell.lassp.houle.RngPack
- uk.ac.standrews.utils.main
- uk.ac.standrews.utils.main.collectionutils
- uk.ac.standrews.utils.main.collectionutils.setutils
- uk.ac.standrews.utils.main.database
- uk.ac.standrews.utils.main.diff
- uk.ac.standrews.utils.main.guiutils
- uk.ac.standrews.utils.main.maputils
- uk.ac.standrews.utils.main.setutils
- uk.ac.standrews.utils.main.stats
- uk.ac.standrews.utils.main.stringutils
- uk.ac.standrews.utils.main.webutils
- uk.ac.standrews.utils.main.webutils.simpledomparser
- uk.ac.standrews.utils.test
- uk.ac.standrews.utils.test.database
- uk.ac.standrews.utils.test.maputils
- uk.ac.standrews.utils.test.setutils
- uk.ac.standrews.utils.test.stats
